# Prevention of Protease-Induced Degradation of Desmoplakin via Small Molecule Binding

**DOI:** 10.3390/jpm14020163

**Published:** 2024-01-31

**Authors:** Isabel M. Romov, Roujon A. Nowzari, Clay P. Page, Madeleine R. Benes, Maegen A. Borzok, Nathan T. Wright

**Affiliations:** 1 Department of Chemistry and Biochemistry, James Madison University, Harrisonburg, VA 22807, USA; 2 Department of Biochemistry, Chemistry, Engineering and Physics, Commonwealth University of Pennsylvania, Mansfield, PA 16933, USA; mborzok@commonwealthu.edu

**Keywords:** desmoplakin, drug screening, calpain, arrhythmogenic cardiomyopathy

## Abstract

Desmoplakin (DSP) is a large (~260 kDa) protein found in the desmosome, the subcellular structure that links the intermediate filament network of one cell to its neighbor. A mutation “hot-spot” within the NH_2_-terminal of the DSP protein (residues 299–515) is associated with arrhythmogenic cardiomyopathy. In a subset of *DSP* variants, disease is linked to calpain hypersensitivity. Previous studies show that calpain hypersensitivity can be corrected in vitro through the addition of a bulky residue neighboring the cleavage site, suggesting that physically blocking calpain accessibility is a viable strategy to restore DSP levels. Here, we aim to find drug-like molecules that also block calpain-dependent degradation of DSP. To do this, we screened ~2500 small molecules to identify compounds that specifically rescue DSP protein levels in the presence of proteases. We find that several molecules, including sodium dodecyl sulfate, palmitoylethanolamide, GW0742, salirasib, eprosarten mesylate, and GSK1838705A prevent wildtype and disease-variant-carrying DSP protein degradation in the presence of both trypsin and calpain without altering protease function. Computational screenings did not predict which molecules would protect DSP, likely due to a lack of specific DSP–drug interactions. Molecular dynamic simulations of DSP–drug complexes suggest that some long hydrophobic molecules can bind in a shallow hydrophobic groove that runs alongside the protease cleavage site. Identification of these compounds lays the groundwork for pharmacological treatment for individuals harboring these hypersensitive DSP variants.

## 1. Introduction

Arrhythmogenic cardiomyopathy (ACM/ARVC) is a rare (1:2000–1:5000) inheritable disease characterized by fibrofatty infiltration of the heart tissue resulting in abnormal heartbeat and impaired cardiac output [1,2,3,4,5,6]. ACM is often diagnosed post mortem, as affected individuals are predisposed to sudden cardiac death, usually in their teens through late 40s [7]. Treatments for those with early clinical signs consist of either the implantation of a defibrillator or the adoption of a largely sedentary lifestyle, along with a cocktail of drugs to minimize cardiac output [6]. There has been a gradual increase in awareness of ACM over the past several decades, due both to a better understanding of the molecular basis of the disease and to increased public awareness [8,9]. Continued advances in our understanding of ACM are critical for eventual treatment; the international task force of ACM states, “the definitive cure of [ACM] will be based on the discovery of the molecular mechanisms that are involved in the etiology and pathogenesis of the disease” [1].

Eighty percent of ACM cases are associated with genes encoding desmosomal proteins [1]. The desmosome is a proteinaceous structure spanning the cell membrane that links the intermediate filament network of one cell to its neighbor [3,10,11]. In the heart, the desmosome is part of the intercalated disk, which also contains adherens junctions and gap junctions [4,11,12,13]. While these three intercellular connections have distinct roles, there is significant interplay between their function and regulation [12,14,15]. For instance, while a weakened desmosome most directly affects how cells relay and disperse physical force through the disruption of load-bearing intermediate filament networks, desmosomal mutations also alter electrical conduction and hamper signal transduction due to a secondary disruption of gap junction localization [16,17,18]. In addition, over time, adipocytes and fibroblasts infiltrate tissues with weakened desmosomes. These two mechanisms are likely the root cause of both arrhythmia and cardiomyopathy [3,4,11,19,20,21].

**Figure 1:**
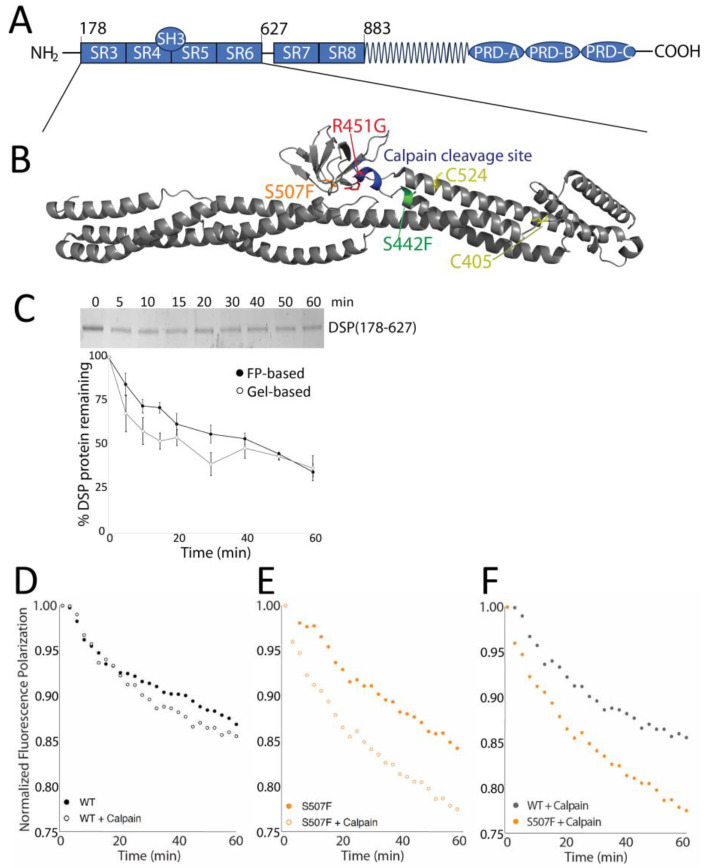
Calpain-hypersensitive desmoplakin mutants can be monitored using fluorescence polarization. (**A**) Schematic of desmoplakin showing the spectrin repeats, the SH3 domain, the central coiled-coil region, and the plakin repeats. (**B**) Structure of sDSP with the position of three variant mutations (S442F, R451G, and S507F), along with the calpain cleavage site and the location of the two exposed cysteine residues. (**C**) Comparison of Coomassie-stained gel and fluorescence polarization-based desmoplakin degradation assays. (**D**–**F**) Example of calpain-induced degradation of WT (**D** + **F**) and S507F (**E** + **F**) sDSP.

About five percent of ACM cases are linked to mutations in desmoplakin (DSP) [1,21,22,23]. The C-terminus of this central desmosomal protein contains three plakin repeat domains that bind to a variety of intermediate filaments (IF), including desmin in the heart (Figure 1A) [10,22,23]. The globular NH_2_-terminus binds to plakoglobin, plakophilin, and the cadherin-like desmoglein and desmocolin, which span the plasma membrane and interact with extracellular regions of desmoglein and desmocolin from the neighboring cell [10,24,25,26]. Adjacent to this protein interaction region is a series of flexible spectrin-like repeats, followed by a long coiled-coil homodimerization domain [27,28].

Most disease-associated DSP variants are located in the plakin repeats and are associated with aberrant IF binding [29,30]. However, a subset of ACM-linked DSP mutations, including R451G, S299R, S442F, and S507F, are located in the spectrin repeat area and are not near any known target interaction regions [17,20,31,32]. Previous studies show that R451G heterozygotes have ~50% lower amounts of DSP in heart tissue, and in vitro studies revealed all of these variants to be hypersensitive to calpain-dependent degradation [17]. In vivo, R451G is homozygous lethal in mice, suggesting a link between DSP levels and disease severity [33]. Molecular dynamics (MD) analysis suggested that this calpain hypersensitivity is due to a weakening of the noncovalent intermolecular interactions surrounding a usually-occluded calpain cleavage site (residues ~447–456) [17,32]. The cleavage site becomes more solvent exposed and thus more readily cleaved in disease-linked variants [17]. A subsequent study demonstrated that this calpain hypersensitivity could be partially prevented through the addition of a bulky amino acid (L518Y) next to the calpain cleavage site [34]. This was postulated to act as a physical barrier that blocks calpain-dependent cleavage.

Here, we explore whether exogenous compounds can also physically block proteases from interacting with DSP variants. With an eye toward an eventual treatment for individuals harboring these mutations, we screened ~2500 FDA-approved drugs and found that select long, hydrophobic compounds can inhibit DSP degradation in vitro without affecting protease function. Molecular dynamics analysis of putative DSP-compound complexes suggest that these drugs bind in a shallow hydrophobic groove neighboring the calpain cleavage site, potentially blocking a productive DSP–protease interaction. While the molecules we identify here likely reside in a poorly defined pocket, the fact that they prevent DSP degradation by binding to a specific area on DSP is foundational for the eventual development of specific molecules that may act as a treatment for select ACM pathologies.

## 2. Materials and Methods

### 2.1. Protein Purification

The human wildtype DSP (residues 178–627) in a pGEX-1 vector construct was a gift from the late Dr. William Weis (Stanford University). Individual variants were generated using Quikchange mutagenesis and verified via whole-plasmid sequencing, as previously described [17]. Plasmids were transformed into *Escherichia coli* (BL21[DE3]), grown in Terrific Broth at 37 °C, and induced with 1 mM IPTG at an OD_600_ of 0.6 overnight at 16 °C. Cells were pelleted, resuspended in phosphate buffered saline pH 7.4 (PBS) with 1–2 mM phenylmethylsulfonyl fluoride (PMSF) and 0.1% Triton-X100, sonicated on ice for 2–4 min in 5 s intervals, and centrifuged at 12,000× *g* for 20 min at 4 °C. The 10–20 mL of resulting supernatant was incubated with 1–2 mL of glutathione Sepharose (Cytiva, Marlborough, MA, USA) on ice for 20 min, and protein was eluted from the beads by incubating 200 U of Thrombin per 2 L of growth for 20 min at 4 °C. Protein was then immediately run over benzamidine resin to remove the thrombin, concentrated, and run over an S200 size chromatography column (Cytiva). Protein purity was determined by SDS-PAGE (12% acrylamide, run for 30 min at 200 V in Tris-Tricine buffer) and concentration via a BCA assay (Thermo; Waltham, MA, USA).

### 2.2. Drug Library

The Cayman chemical “FDA-approved drug screen” (Cayman Chemicals; Ann Arbor, MI, USA) was used as the drug library. A total of 2496 10 mM drugs dissolved in DMSO were stored in 27 96-well plates at −80 °C until use. All fluorescent and red/yellow-colored compounds were removed before testing.

### 2.3. Fluorescence Polarization Assays

DSP_178–627_ (sDSP) at 1 mg/mL was fluorescently labeled with a 10-fold excess of Fluorescein-5-malemide (Thermo) overnight at 4 °C and passed through a G25 Sepharose (Cytiva) column to separate labeled protein. Labeling efficiency was calculated following the vendor’s instructions and tended to be ~1.5 FITC labels per DSP_178–627_ monomer.

Fluorescence Polarization (FP) assays were conducted in 396-well format using a BioTek Synergy H4 plate reader with polarization capacity (Agilent; Savage, MD, USA). All samples were run at 25 °C using PBS pH 7.4, 1 mM DTT, 10 mM CaCl_2_, 5% DMSO, and 1 mM FITC- DSP_178–627_, with 500 μM of each drug. Trypsin, chymotrypsin, or calpain at either 1:50, 1:50, or 1:20 concentration was added directly before data collection with an average void time of 2 min. Control experiments in each plate were conducted in triplicate and consisted of DSP_178–627_ + DMSO + protease as a positive control and DSP_178–627_ + DMSO + PMSF + protease as a negative control. Only plates that had functional positive and negative controls were used in data analysis. Samples were excited at 485 nm, and polarization was monitored at 528 nm. Data were collected at 5 min increments for 1–3 h.

### 2.4. Calpain Assays

Calpain assays were performed as previously described with minor modifications [34]. A total of 5–20 μg of purified recombinant DSP_178–627_ protein were added to PBS pH 7.4, 10 mM CaCl_2_, and 1 mM DTT at room temperature. Protease was added at a 1:20 ratio and quenched in 1% SDS at 95 °C. Quantification of the total amount of protein remaining was calculated via densitometry obtained from ImageJ software version 2.9.0 (NIH). Values are the average of at least four independent replicates.

### 2.5. Molecular Dynamics

Computational modeling was performed using the human wildtype DSP_178–627_ (PDB accession 3R6N) [28]. SMILES files derived from the drug library were converted into scene objects in the MD program YASARA using an AMBER14 forcefield, and then docked, using the VINA docking macro, into a 3 nm^3^ region that included the DSP calpain cleavage site, the DSP hydrophobic cleft, and roughly 1 nm of neighboring protein [35]. MD simulations of DSP_178–627_-drug combinations were run with wildtype DSP_178–627_ in duplicate for an average of 100 ns in explicit solvent at 37 °C, 150 mM NaCl, as previously described [17]. Simulations were surveyed every 100 ps, and the RMSF and drug–protein distances were tabulated using YASARA macros.

### 2.6. Statistics

Data are represented by the mean and SEM. Data were compared via ANOVA tests. To control for large data sets, multiple comparisons using a random array of degradation data were done with 1-way ANOVA tests to determine statistical significance. *p* values of less than 0.05, indicating a 95% or greater confidence level, were considered significant.

## 3. Results

### 3.1. Fluorescence Polarization Assays Can Monitor DSP Degradation

Previous studies that identified and characterized the series of calpain hypersensitive DSP variants associated with ACM did so using the NH_2_-terminus of human DSP (a construct containing amino acids 1–883, Figure 1A) [17,34]. Poor protein expression and instability hampered large-scale studies using this construct, and so here we used the shorter DSP construct (DSP_178–627_), henceforth referred to as sDSP. This fragment has been crystalized (PDB: 3R6N; [28]), can be expressed at much higher amounts (~2 mg/L vs. ~0.1 mg/L), and is more amenable to long-term storage. Each of the ACM-linked variants associated with calpain hypersensitivity (S299R, S442F, R451G, and S507F), along with the calpain cleavage site (Q447-R456), are located within this construct, near the interface between SR4, SR5, and the SH3 domain.

Previous studies assessing DSP stability have relied on gel-based assays, which are both time- and resource-intensive [17,34]. We therefore developed a fluorescence polarization (FP) assay to quickly screen DSP stability over a large number of conditions. sDSP contains only two exposed cysteine residues (Figure 1B) that can be FITC-labeled. These cysteines are ~2 and 5 nm away from the calpain cleavage site, respectively. To test whether FITC-labeled sDSP could be used to monitor calpain-dependent degradation, we compared gel-based and FP assays on sDSP and FITC-labeled sDSP, respectively (Figure 1C). The densitrometry from the gel-based assay and FP data are similar, although the FP data tend to indicate marginally slower degradation at early timepoints. We thus conclude that, using these conditions, FP polarization data are comparable to gel-based assays at time points after 20 min. We used the 60 min timepoint for all future experiments.

Based on our previous results, DSP_1–883_ variants hyperdegrade in the presence of calpain while wildtype DSP_1–883_ shows only minimal degradation [17]. To test if FITC-labeled sDSP behaved similarly, we subjected wildtype and variant sDSP to calpain as previously described. All variants displayed basal level of degradation in our assay, likely due to either normal DSP protein denaturation that takes place at room temperature and/or trace amounts of contaminating bacterial proteases; addition of protease inhibitor PMSF largely prevented this degradation. When calpain is added, sDSP wildtype degrades at a similar rate to previous reports using DSP_1–883_ (Figure 1D) [17]. sDSP-S507F both exhibits increased rate of basal degradation and is more susceptible to calpain-specific degradation (Figure 1E,F), also in agreement with previous data. Thus, this new experimental system is comparable to previous methods.

### 3.2. Proteases Other Than Calpain Can Hyperdegrade DSP Variants

**Figure 2:**
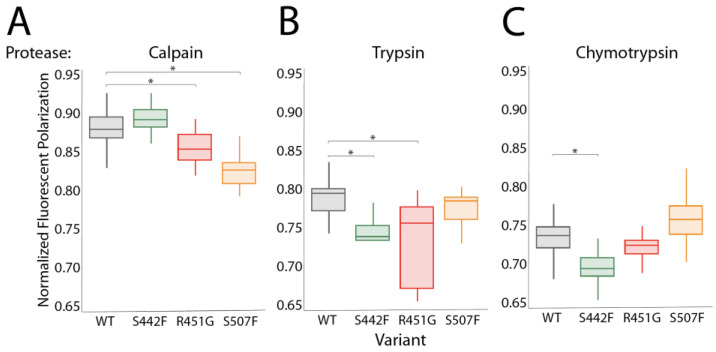
Desmoplakin mutants are sensitive to a variety of proteases. (**A**) Calpain degrades DSP_178–627_ R451G and S507F faster than WT. (**B**) Trypsin degrades DSP_178–627_ S442F and R451G faster than WT. (**C**) Chymotrypsin degrades DSP_178–627_ S442F faster than WT. Asterisks indicate significance (*p* < 0.05).

The DSP calpain cleavage site is protected by a web of stabilizing hydrogen bonds and electrostatic interactions that anchor the site to the rest of the molecule. This web in DSP variants is compromised, resulting in a localized loss of stability and subsequent solvent exposure of the cleavage site [17,34]. Figure 1D,E suggest that sDSP variants may also be sensitive to proteases other than calpain. In support of this, variant constructs have lower protein purification yield than wildtype and must be grown at lower temperatures despite previous reports showing that none of these variants alter the DSP T_m_ [17,34]. The calpain cleavage site (447-QLKPRNPDYR) contains both potential trypsin and chymotrypsin sites (in bold), and so we suspected both proteases would also preferentially cleave DSP variants. To test whether DSP degradation is calpain-specific, we incubated wildtype and variant sDSP with calpain, trypsin, or chymotrypsin (Figure 2A–C). S299R sDSP could not be purified without degradation, and thus all subsequent experiments were conducted on WT, R451G, S507F, and S442F constructs. The R451G and S507F variants are hyperdegraded in the presence of calpain. Interestingly, the S442F variant degrades at roughly the same rate as WT in the presence of calpain, possibly due to interference from the FITC tag, which is only ~1 nm from the mutation site. The S442F and R451G variants are hypersusceptible to trypsin cleavage while the S507F variant is not. S442F but not R451G or S507F cleave more readily than wildtype in the presence of chymotrypsin. Thus, all variants are sensitive to a unique combination of proteases. It is worth noting that other types of proteases could potentially target different cleavage sites on DSP than the ones tested here.

### 3.3. Many Drugs Can Prevent DSP-Specific Degradation In Vitro

**Figure 3:**
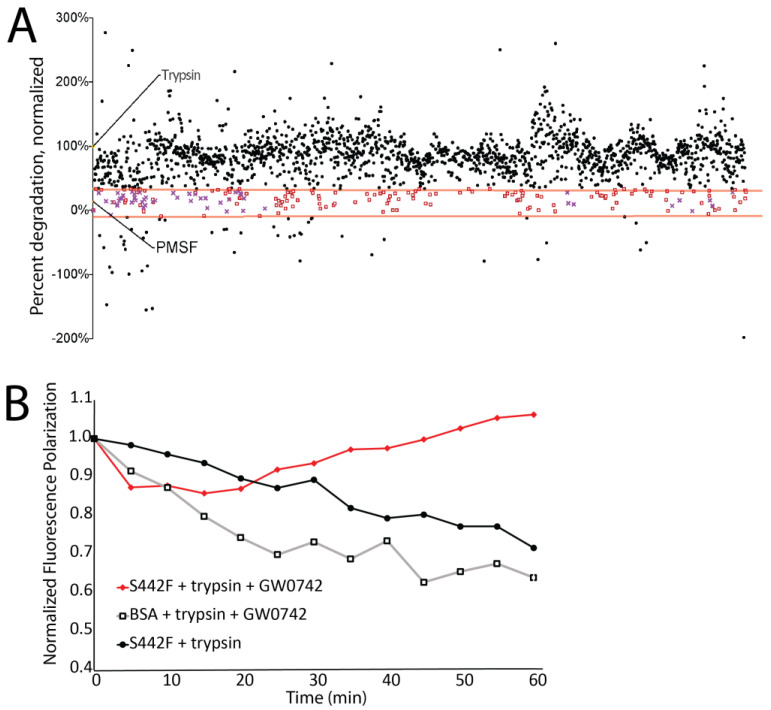
Compounds inhibit desmoplakin degradation. (**A**) sDSP S442F degradation in the presence of trypsin plus 2496 compounds from the Cayman chemical “FDA library”. A degradation rate of 100% indicates the same desmoplakin degradation rate with and without the addition of 500 μM drug while a 0% rate indicates no degradation, as judged by fluorescent polarization values after 1 h. A trypsin-only control and a trypsin + PMSF control is indicated. Protection (red lines) was arbitrarily set between 33% and −10%. Compounds that protected desmoplakin from degradation are shown in red boxes, and compounds that additionally showed no protection against trypsin digestion of BSA are shown in purple Xs. (**B**) Example of how the data in A was generated. Desmoplakin + trypsin, and BSA + trypsin + GW0742 degrade over one hour, while desmoplain + trypsin + GW0742 does not degrade.

A mutation that partially occludes the DSP calpain cleavage site rescues DSP protein levels [34]. We next assessed whether protease-dependent cleavage can also be blocked by small molecules. We screened 2496 FDA-approved drugs for their ability to prevent trypsin-dependent cleavage of sDSP S442F in our FP assay (Figure 3A). These data were normalized to trypsin-treated sDSP S442F, set at a degradation rate of 100%. No change in fluorescence polarization after an hour is indicative of 0% protein degradation rate, as occurs in the PMSF positive control. This screen identified ~250 small molecules that prevented sDSP S442F degradation (Figure 3A, red squares).

Examination of the top 250 molecules revealed many to be protease inhibitors. Using protease inhibitors as an eventual therapeutic strategy is undesirable due to the strong likelihood that such inhibition would have deleterious off-target effects. To identify compounds that protect DSP without influencing protease function, we next performed a secondary screen to assess which molecules prevented degradation of a non-specific FITC-BSA target. In total, 62 compounds both prevented sDSP S442F degradation and did not prevent BSA degradation (Figure 3A; purple X’s; Figure 3B; Supplemental Table S1; Supplemental Figure S1).

**Figure 4:**
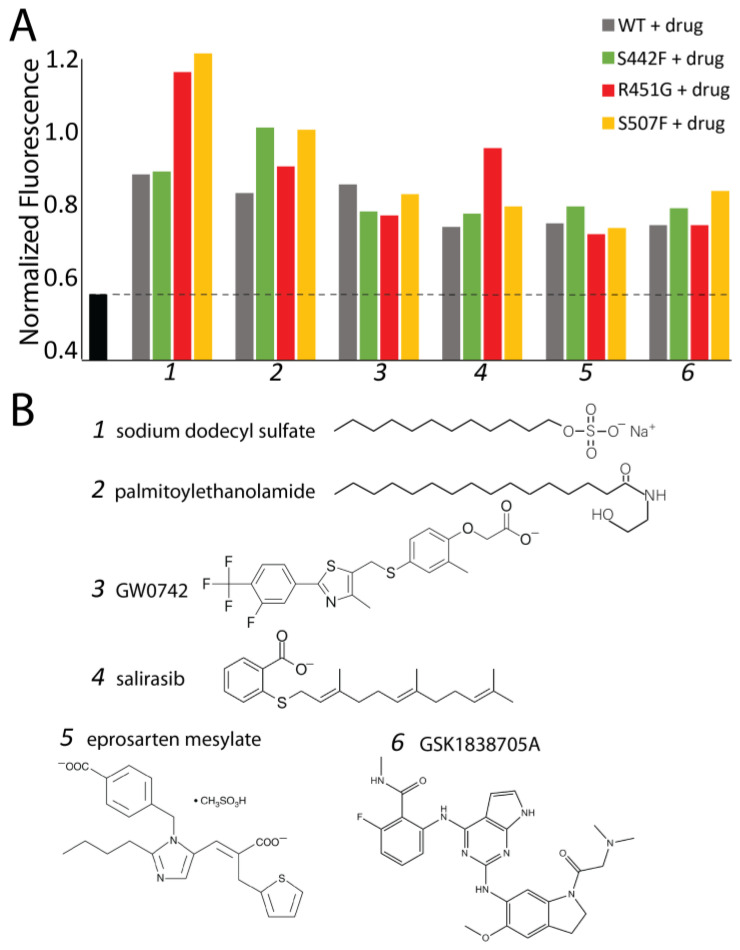
Drugs that inhibit both WT and variant sDSP degradation. (**A**) Normalized fluorescent polarization of WT and sDSP variants in the presence of a 1:20 ratio of calpain plus 500 μM drug, as compared to calpain alone (black) (**B**) Identity and chemical structure of the six best-performing compounds.

These screens were conducted using trypsin because of its accessibility and ease to work with; however, while our data showed that trypsin and calpain act similarly on sDSP, they are not equivalent. Therefore, we next tested whether these 62 compounds also prevent wildtype and variant DSP degradation in the presence of calpain. Six compounds protected all four DSP constructs from calpain degradation (Figure 4A). An additional three compounds protected three of the four proteins tested, and eleven more compounds protected two of four sDSP proteins (Supplemental Table S2).

### 3.4. Computational Screening Identifies a Shallow Hydrophobic Cleft Where Drugs May Bind

**Figure 5:**
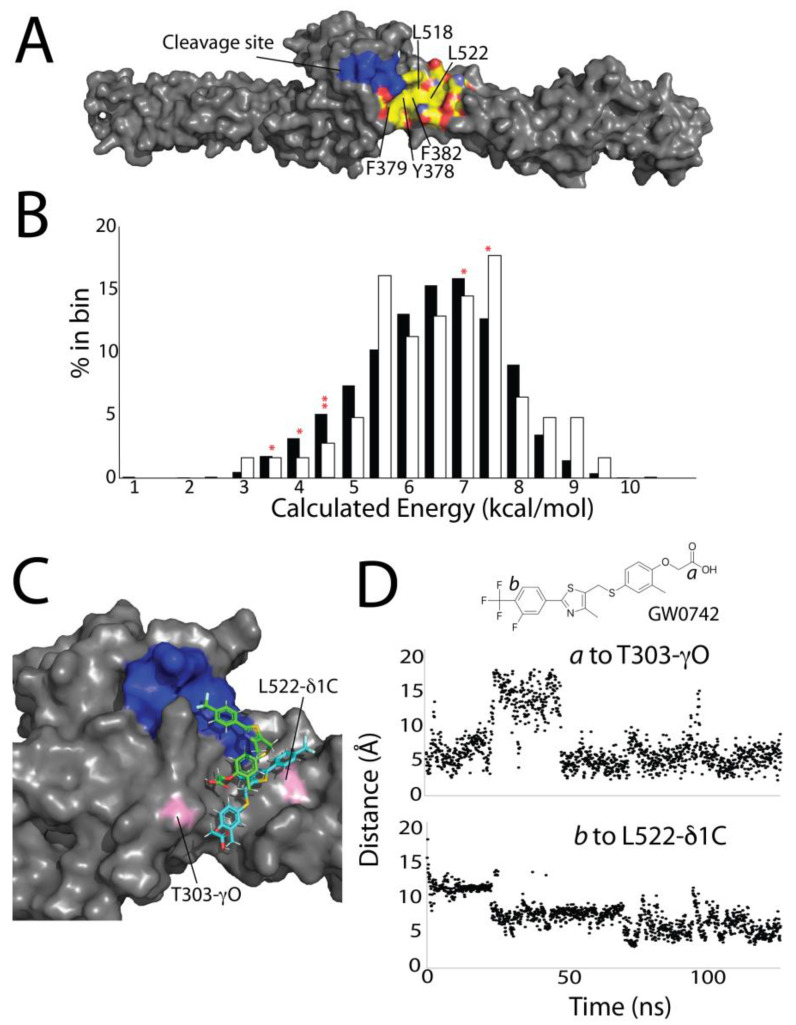
Computational screening does not predict drug efficacy. (**A**) Surface map of the protease cleavage site (blue) and the hydrophobic groove (hydrocarbons are colored yellow, oxygens are colored red, nitrogens are colored light blue). (**B**) Percentage of drugs in the overall screen (black) and the top 62 best molecules (white) sorted by calculated binding energy. Red asterisks denote the energies of the top six compounds. (**C**) Example of the motion of a small molecule in a VINA-docked structure (green) and after 100 ns MD simulation (teal). (**D**) Visualization of simulated GW0742 molecular motion in the hydrophobic cleft, measured as a distance to T303 to carbon *a* (top) or L522 to carbon *b* (bottom).

The top six compounds have several commonalities. All contain at least 12 carbons and are predominantly hydrophobic. Four contain carboxylates, and three are fatty acid derivatives (Figure 4B). Since these compounds influence DSP degradation specifically, they likely bind to a DSP hydrophobic region near the protease cleavage site. To identify this site, we used a VINA computational docking protocol, where we defined the drug interaction area to be anywhere within 6 nm surrounding the protease cleavage site (Supplemental Figure S2). This screen suggests that binding occurs at one of two areas: either in a cavity between the distal interface of the SH3 domain and SR4, or in a hydrophobic groove on SR5 neighboring the protease cleavage site. We reasoned that this second site, consisting of residues Y378, F379, F382, L518, and L522 lined with the polar or charged residues D300, Q386, E389, K525, and D521 (Figure 5A), was more likely due to its proximity to the cleavage site.

To more fully probe the binding characteristics of this hydrophobic groove, and to test how well computational screening techniques align with our experimental results, we computationally docked the same ~2500 compounds we had tested experimentally into this hydrophobic groove using VINA AutoDock [36]. From these results, we extracted the theoretical binding energies (Figure 5B) and compared these values to the experimental inhibition values (Figure 3A). This computational approach showed no difference in binding energies of our top 62 experimental compounds for DSP as compared to the entire drug library (Figure 5B). Virtually no drugs (<100) could have been culled from the original drug library without removing some of the top 62 experimentally determined compounds. Thus, this theoretical binding calculation alone is not predictive of in vitro drug efficacy in this situation. Four of the top six experimental compounds had computationally determined binding energies much below the average of 6.2 kcal/mol (red asterisk in Figure 5B). Given the wide and shallow nature of the hydrophobic pocket, we hypothesize there is not a sufficiently large or specific interaction surface to generate robust predictive data computationally.

While the computational screen did not predict drug efficacy, we also assessed the model’s ability to predict the position of the small molecule in the hydrophobic pocket. In MD simulations of the top six experimental drug/DSP complexes, the small molecules remained bound to DSP S442F for the duration of all simulations (2×~100 ns for each complex). However, all small compounds moved around significantly within the hydrophobic groove (Figure 5C,D, Supplemental Figure S3). This, likely, stems from the same principles that contribute to the low binding energy; while there is a large hydrophobic region to which small molecules can bind, it is shallow, wide, and does not have many polar or electrostatic moieties to anchor specific drug interactions.

## 4. Discussion

Previous work has shown that S442F hyperdegrades in the presence of calpain, yet we did not see this effect in our system [17,34]. The most likely explanation for this discrepancy is our use of the FITC label on exposed cysteines, C524 and C405. C524 is on the periphery of the hydrophobic groove, and as subsequent data modeling data show (Figure 5), the inclusion of a moderately hydrophobic moiety in this region may affect protease-specific DSP degradation. S442F is only ~1 nm from C524, and thus the structural changes brought about by S442F may be more susceptible than other variants to an influence by the FITC label. Although our FITC-labeling protocol proved robust, future work should substitute serine for cysteine at this site to simplify and standardize data analysis.

We show that DSP variants are sensitive in our in vitro assay to proteases other than calpain. This is not surprising; the variants expose the protease cleavage site to the surrounding solvent. Despite this finding, it remains likely that calpain is the cellular protease responsible for DSP degradation in ACM. Trypsin and chymotrypsin are not expressed in myocardia. Of the proteases that are expressed in the heart, matrix metalloproteins (MMPs), caspases, cathepsins, and calpains have been broadly implicated in heart diseases [12,37,38]. sDSP does not contain the target sequence for any caspase [39]. Cathepsins are almost exclusively active in the lysosome [40,41]. The consensus recognition site of MMPs is poorly defined, but sDSP is not a likely target [42,43]. In contrast, the blocking of calpain activity has been shown experimentally to increase R451G DSP levels [17]. Thus, our description of trypsin-dependent DSP cleavage should be interpreted merely as a convenient in vitro proxy for calpain sensitivity, and not as a physiologically relevant mechanism of DSP degradation.

About 50 compounds showed upwards slopes (and thus negative degradation percentages) in our degradation assay, suggesting that the size of DSP was increasing with time. The most likely explanation of these data is that the agent induced protein aggregation. Future tests using dynamic light scattering can verify this hypothesis. Another possibility is that these compounds change the viscosity of the solution sufficiently to alter the fluorescence polarization; however, we noticed no difficulty in pipetting any of these compounds, and the FP profiles continued to increase over several hours which is inconsistent with a simple viscosity change. Conversely, multiple compounds caused faster degradation than when trypsin alone is added (Supplemental Figure S4). We hypothesize these compounds destabilize or unfold DSP or increase affinity of the protease to DSP interaction through some unknown mechanism.

VINA docking does not effectively predict which compounds prevent DSP degradation. This is likely due to the paucity of drug–protein interactions, brought about by the shallowness of the hydrophobic groove. While this aspect of VINA docking likely explains why it was not predictive, it does not explain why the best computational hits were also not experimental hits. This is likely for two reasons. First, the experimental drug screen was conducted at a 500 μM of compound, which may allow for the selection of low-affinity interactions. Second, an examination of the top computational hits suggested that our computational screen emphasized a different set of factors than our in vitro screen. Of the top 15 computational hits, 5 were insoluble in water and thus likely precipitated in the FP polarization assay. Four were colored. A total of ~200 compounds were removed from experimental testing due to the drugs possessing a color, which interferes with the FITC FP assay. Three of the computationally docked drugs were in chemically unlikely orientations. For instance, two had negatively charged phosphate groups on separate arms of the small molecule in close proximity. Of the three remaining compounds, two were part of our 62 best hits and one was not. This exercise underscores the importance of experimentally validating computational data; computational screens do not always consider experimental realities.

## 5. Conclusions

Here, we identify compounds that specifically prevent DSP degradation in the presence of several proteases. These compounds do not affect the function of the protease and therefore likely bind to wildtype and variant sDSP by blocking protease access to the protein. Drug–DSP binding likely occurs at a hydrophobic groove neighboring the protease cleavage site. Due to the size and shallowness of this site, computational screening methods were not useful in identifying the most likely hits, but MD did produce reasonable models that visualize how compounds fit into this hydrophobic groove and prevent proteolysis. In total, this work lays the groundwork for future studies that more fully characterize DSP–small molecule interactions. The long-term goal remains to develop compounds that can specifically bind and stabilize DSP and DSP variants, thus preventing desmosome-related diseases.

## Data Availability

All data can be obtained by contacting N.T.W. (wrightnt@jmu.edu).

## Supplementary Materials

The following supporting information can be downloaded at: https://www.mdpi.com/article/10.3390/jpm14020163/s1, Figure S1: Example of a drug that rescued 2 of 4 DSP variants in the presence of calpain (CCT128930); Figure S2: YASARA scene showing sDSP and the initial box size where drugs dock in the VINA program; Figure S3: Simulation over 100 ns of the top 6 compounds showing their degree of motion in the DSP hydrophobic groove; Figure S4: The identity and structure of compounds that at least doubled the DSP degradation rate; Table S1: 62 best hits, with their normalized degradation rates for DSP and BSA; Table S2: Drugs that inhibit trypsin degradation of DSP S442F, do not inhibit BSA degradation, and inhibit the degradation of at least 2 DSP variants in the presence of calpain.
