# Supplementary material for: Prevention of Protease-Induced Degradation of Desmoplakin via Small Molecule Binding
Source: J Pers Med. 2024 Jan 31;14(2):163. doi: 10.3390/jpm14020163 (PMC10890502; doi:10.3390/jpm14020163)
Supplement: Supplementary file 1 [file jpm-14-00163-s001.zip › Supplemental Tables.pdf]

Supplemental Table S1: 62 best hits, with their normalized degradation rates for DSP and BSA.

Drug Name	Normalized DSP degradation rate	Normalized BSA degradation rate
2-Cl-IB-MECA	5	89
Adapalene sodium salt	15	101
Amikacin	21	100
Amitriptyline HCl	13	107
AT7867	31	123
AZD3759	33	86
Benidipine HCl	15	80
Betamethasone Valerate	19	111
Bexarotene	26	100
CCT128930	20	103
Cinacalcet HCl	16	106
Citric acid	32	113
Clomiphene citrate	23	89
Desonide	-8	80
Diosmetin	18	98
Docosahexaenoic Acid	32	99
Doramectin	4	99
Dronedarone	-4	103
Dronedarone HCl	23	124
Eprazinone 2HCl	5	82
Eprosartan Mesylate	32	90
Fendiline (hydrochloride)	29	107
Fingolimod (FTY720)	-1	89
Flubendazole	-1	100
Gliquidone	29	114
GSK1838705A	32	80
GW0742	6	119
Ibandronate sodium	19	86
Imatinib (STI571)	-3	80
IMD 0354	23	95
JNJ-38877605	18	79
Latanoprost	9	84
LDE225 Diphosphate	5	81
Lithocholic Acid	2	101
Lomitapide	32	87

Loxapine Succinate	1	87
LY2584702	18	126
LY2608204	12	101
LY335979 (Zosuquidar 3HCL)	29	86
Mevastatin	26	81
Neomycin sulfate	24	82
Orlistat	23	88
Oxybutynin chloride	24	99
PAC-1	31	120
Paclitaxel (Taxol)	31	80
Palmitoylethanolamide	26	80
Paroxetine HCl	20	129
Pergolide mesylate	20	89
Perifosine	8	84
Phenformin HCl	20	85
PHT-427	28	88
Reserpine hydrochloride	31	98
RG7112	3	83
RG7388	-3	87
Romidepsin (FK228, depsipeptide)	-2	96
Saikosaponin A	10	93
Salirasib	-8	106
Sodium Aescinate	19	87
Sodium dodecyl sulfate	18	93
Sulconazole Nitrate	21	87
Ticagrelor	0	80
Tizanidine HCl	27	94

Supplemental Table S2: Drugs that inhibit trypsin degradation of DSP S442F, do not inhibit BSA degradation, and inhibit the degradation of at least 2 DSP variants in the presence of calpain.

**Drug**	**Normalized DSP screen**	**Normalized BSA screen**	**WT-calpain normalized rate**	**S442F-calpain normalized rate**	**R451G-calpain normalized rate**	**S507F-calpain normalized rate**
**GW0742**	**6**	119	**21**	**35**	**38**	**24**
**Eprosarten Mesylate**	**32**	90	**30**	**26**	**33**	**31**
**SDS**	**18**	93	**24**	**21**	**-20**	**-30**
**GSK1838705A**	**32**	80	**35**	**23**	**34**	**24**
**Salirasib**	**-8**	106	**32**	**29**	**-5**	**26**
**Palmitoylethanolamide**	**26**	80	**22**	**-3**	**8**	**3**
**Paraxetine**	**20**	129	**-8**	**-3**	26	133
**Dronedarone HCl**	**23**	124	143	**0**	**20**	**25**
**Sodium Aescinate**	**19**	87	**-5**	**13**	**11**	99
**Loxipine Succinate**	**1**	87	**-8**	101	**-41**	107
**CCT128930**	**20**	103	54	86	**32**	**20**
**Taxol**	**31**	80	48	87	**38**	**34**
**Bexarotene**	**26**	100	81	84	**39**	**4**
**Lomitapide**	**32**	87	118	62	**40**	**4**
**Diosmetin**	**18**	98	84	73	**30**	**0**
**Oxybutynin chloride**	**24**	99	91	**-5**	**31**	77
**Imatinib**	**-3**	80	**-6**	62	**4**	108
**LY2584702**	**18**	126	67	**-40**	**15**	84
**Fingolimod**	**-1**	89	**35**	70	**-13**	**32**
**Docosahexaenoic Acid**	**32**	99	**-12**	**-19**	118	104

Key

Inhibits 4 of 4 DSP variants

Inhibits 3 of 4 DSP variants

Inhibits 2 of 4 DSP variants

bold denotes slower degradation than protease alone
