# Supplementary material for: Prevention of Protease-Induced Degradation of Desmoplakin via Small Molecule Binding
Source: J Pers Med. 2024 Jan 31;14(2):163. doi: 10.3390/jpm14020163 (PMC10890502; doi:10.3390/jpm14020163)
Supplement: Supplementary file 1 [file jpm-14-00163-s001.zip › Supplementary Figures.pdf]

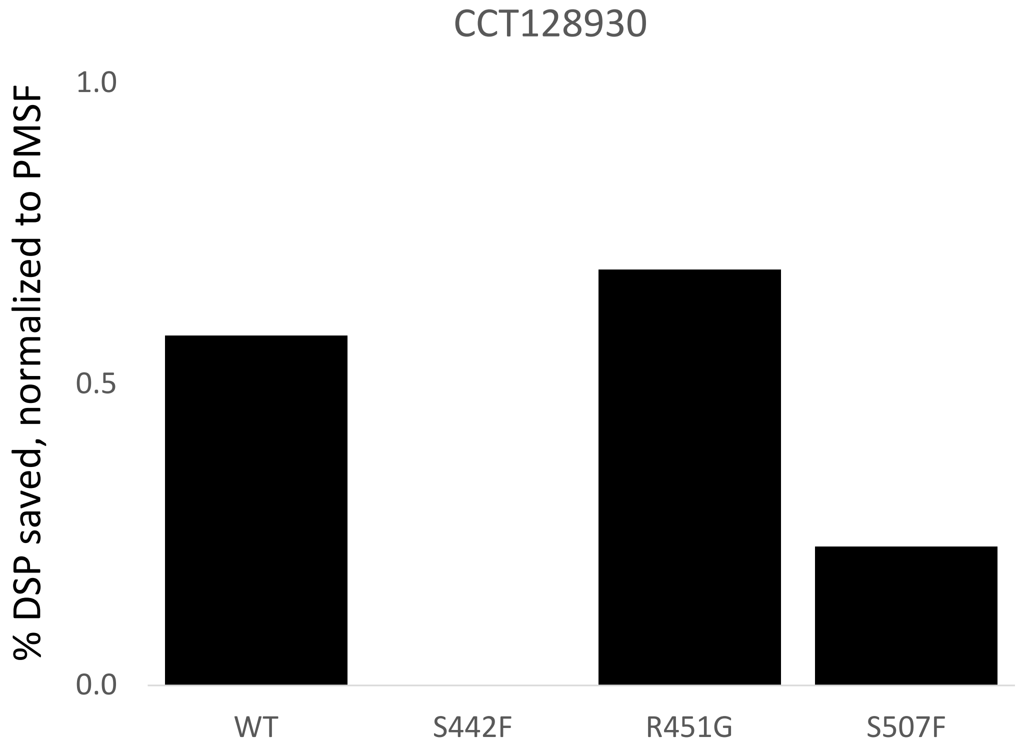

Figure S1: Example of a drug that rescued 2 of 4 DSP variants in the presence of calpain (CCT128930). WT and R451G were rescued (~55% and 80%) while S442F showed 0% rescue and S507F showed ~25% rescue in the presence of the drug.

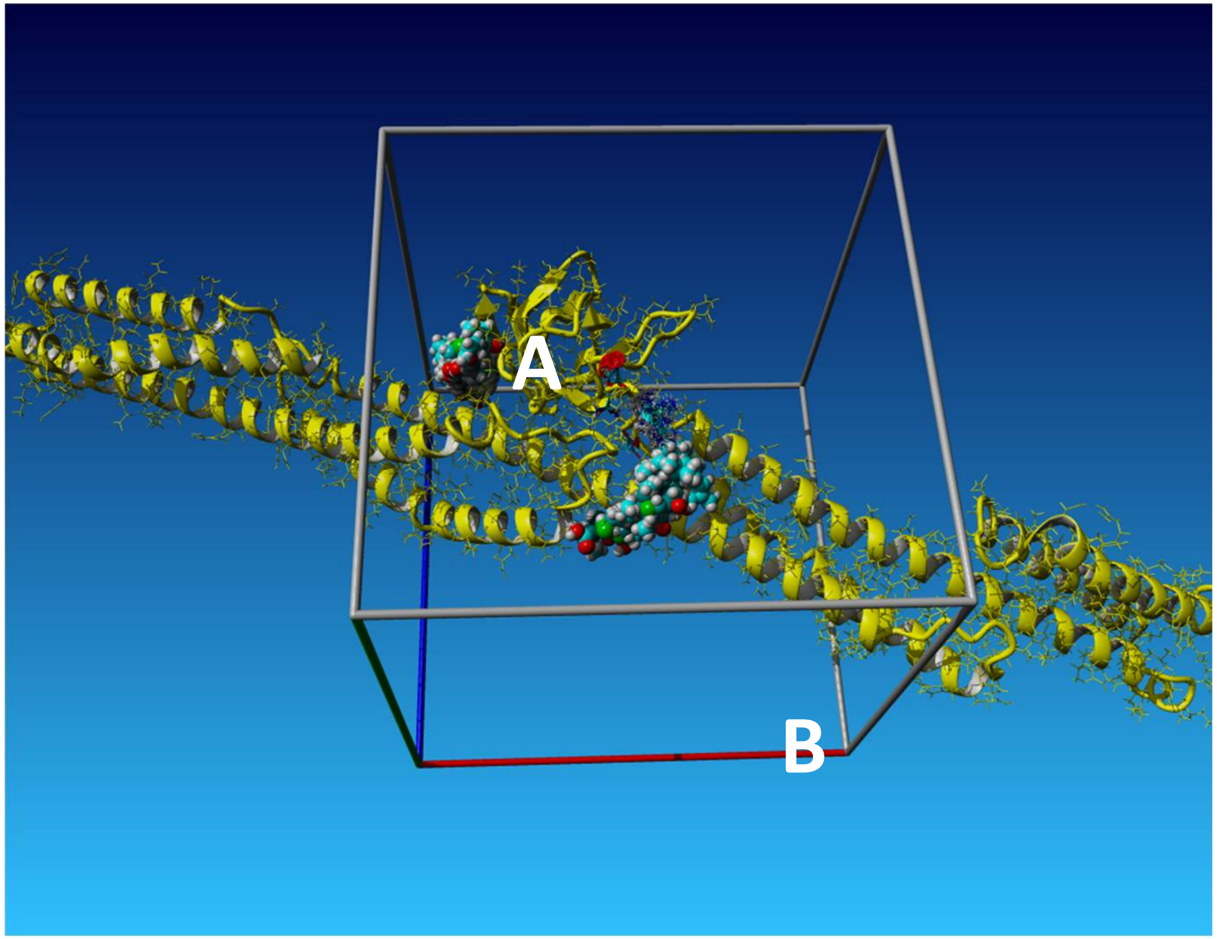

Figure S2: YASARA scene showing sDSP and the initial box size where drugs dock in the VINA program. A is the distal region discussed in the next. B is the hydrophobic groove, and the site ultimately chosen for the more extensive docking, given its proximity to the calpain cleavage site (shown in colored sticks near the middle of the box).

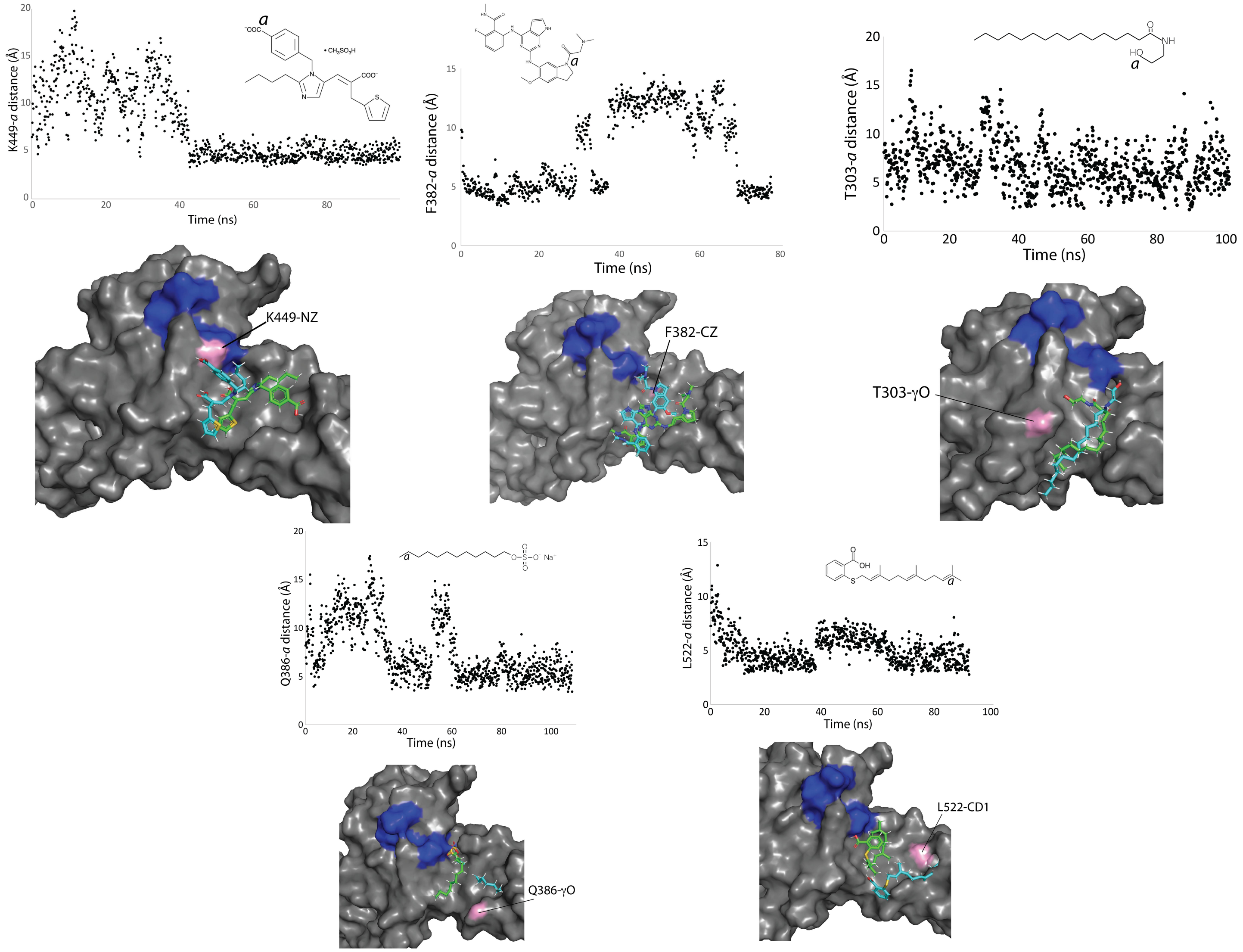

Figure S3: MD simulations of the other best 6 compounds. In each, green represents the drug position after VINA docking, and blue represents the

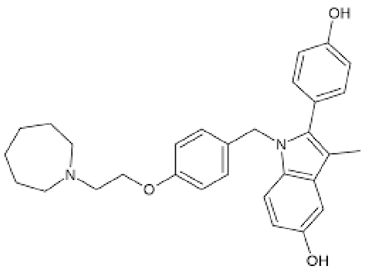

Bazedoxifene acetate: 330%

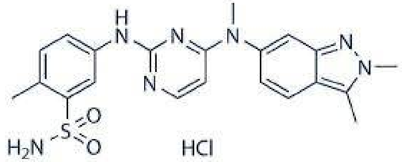

Pazopanib: 340%

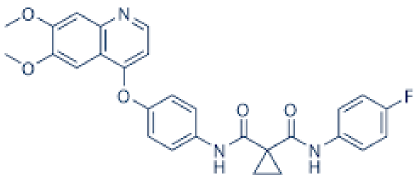

Cabozantinib: 330%

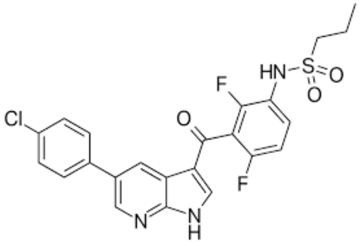

Vemurafenib: 325%

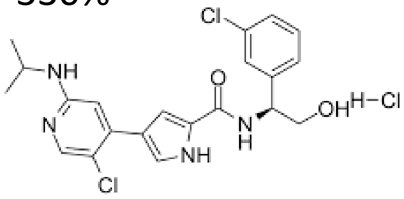

Ulixertinib: 275%

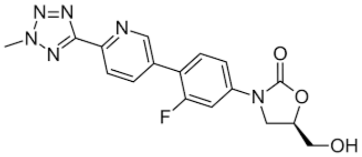

Tedizolid: 275%

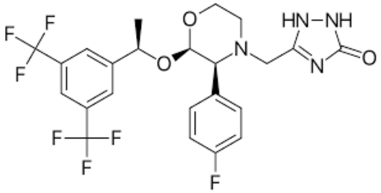

Aprepitant: 275%

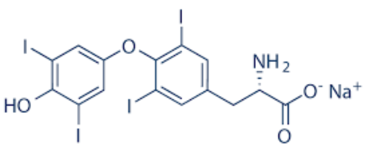

Levothyroxine: 250%

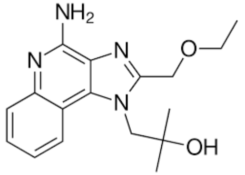

Resquimod: 240%

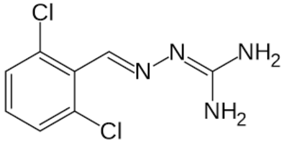

Guanabenz: 230%

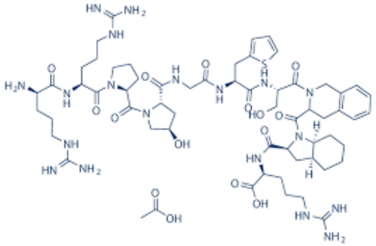

Icatibant Acetate: 225%

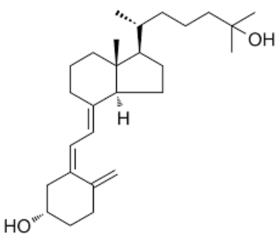

Calcifediol: 215%

Figure S4: Drugs that degrade DSP more than 2x faster than with trypsin alone.
